# Surgical Diseases

**Published:** 1863-05

**Authors:** 


### SURGICAL DISEASES.

MULTITUDES perish prematurely, or suffer dreadful agonies for years, in consequence of neglecting slight deformities in the body, or swellings or protrusions, which, although they may give little or no pain, do nevertheless sometimes lead to deplorable results; hence it is thought a public benefit may result from making some plain statements in reference first to ailments which may require surgical treatment. It is a common but very erroneous opinion that surgical interference must necessarily be accompanied with pain and danger to life; that the knife must be used and blood must flow. The fact is, that in the hands of a skillful surgeon, the cases are rare which require any thing more than a very small amount of heroism to bear. In cases where the patient is of a very nervous temperament, a slight inhalation of ether or other anæsthetic will render him insensible to pain during the very few moments of the operation. In the majority of surgical operations, in ordinary practice, there is a lessening of pain from the instant of the first touch of the instrument, especially such as are required for strictured passages, displacement of various organs, the application of topical remedies, and the adaptation of the various kinds of mechanical support, and the like.

Surgery is a science, hence its practice is not uncertain. It is based on physiological law; it is an art founded on anatomical knowledge, and experience in the use of proper instruments and remedies. The success of a surgical operation can be predicted with great confidence if properly performed, provided the vital status is not too much impaired by extraneous or unforeseen circumstances, such as great loss of blood or nervous depression from some terrible injury, or exhaustion from some wasting or malignant disease.

The writer's labor as a practical anatomist during many years, and his experience in surgical diseases, authorize him to speak and act with authority in the premises; and first as to

### TUMORS IN GENERAL.

If a tumor is taken from the body, "surgical repair" is necessary to a perfect cure; this is a process by which lymph, or the fibrous portion of the blood, is thrown out and wakes into life, becomes a part of the living body, and by this means all wounded or fractured parts are repaired and become united, and cavities are filled up and obliterated.

A TUMOR is a preternatural eminence existing in any part of the body; when external, it is more or less a deformity; very often painful, and sometimes inconvenient, especially when large. Sometimes after remaining inactive for years, they suddenly become malignant and prove speedily fatal. A tumor may be transient, as when caused by effusion or inflation; these the skillful surgeon will remove by promoting their absorption, or will otherwise cause them to disappear without violence; the charlatan or the youngster removes them with the knife or terribly burning applications, and then boasts of having cured cancer or malignant tubercle.

PERMANENT TUMORS may be caused by the impaction of a foreign substance, by some unnatural growth of parts or organs, deposits of water or blood, of calcareous and other matter; by hernia, by the dilation of large vessels, as aneurisms. They may be of all sizes, from a wart to a mass equaling in size the whole body; they may be cancerous or otherwise extremely dangerous. When there exists the slightest doubt as to their true character, an honorable, conscientious, and experienced surgeon should be promptly consulted. Many, very many cases are on record, where incalculable mischief has been done by ignorant interference. A case: In July, 1859, the writer was called upon by a distinguished Philadelphia surgeon to examine a tumor on the back of a lady. It extended from the neck to the loins, forming a most unsightly hump. It was at once pronounced an encysted tumor, and its immediate removal advised, not only on account of the deformity, but because it was on the point of ulcerating, which event would have given rise to a most offensive discharge, which might have continued for a lifetime. Although nursing a child at the time, the lady consented to an operation, which the writer performed, without administering ether, and with but little pain. The tumor weighed four pounds. The patient never kept her bed; resumed her usual active duties within a fortnight, well pleased with being rid of such a superfluous burden with but really a slight inconvenience.

It must not be inferred that all tumors must be removed with the knife. Many can be removed by reäbsorption, some by tapping, and others, as aneurisms, by tying a string around a proper blood-vessel.

### FACE DEFORMITIES.

Sometimes persons are born with a want of the features, with deformities in the skin, such as "mother's mark," (aneurism by anestimosis,) moles, and the like. Deformities may arise after birth from disease or injury, such as pitting from small-pox, scars from scrofulous abscess, burns, scalds, cuts and fistula; then, again, there is a loss or deformity of features by disease or violence.

### MOTHER'S MARK.

When small, or of a form which facilitates removal, it is readily removed, and should be done as soon as possible in infants and young children, in order to prevent increase in size.

Very often after the healing of an abscess, there remains an unsightly puckering of the skin. In almost all such cases a portion of the surplus skin can be removed with great nicety.

### BURNS.

When the skin has been destroyed by a burn, the wound contracts during the process of repair, and draws the adjacent skin with it, as in pulling down the side of the mouth or eyelids, or exposing more or less of the mucous membrane of the lower eyelid, which, by dust lodging on it, and by the constant change of temperature, a permanent source of irritation is set up, endangering vision.

In extensive burns about the neck, the skin contracts so much that the lower jaw is drawn down to the extent of preventing the mouth from closing completely, causing a hideous appearance, while the constant dribbling of saliva from the corners of the mouth is an incessant and mortifying annoyance. Here defects, large and small, can be remedied by proper operative procedure, and moles, hair, discolorations of the skin, and other unsightly appearances can be removed without danger or suffering, scarcely leaving the slightest trace of their previous existence; and yet there are multitudes of parents who, under the impression that these things can not be remedied, allow their children to grow up with these blemishes, which in too many cases is a life-long martyrdom to them from their constantly growing sensitiveness in relation to them. Such a result is greatly to be deplored, and many a heart-ache may be prevented as to a lovely daughter, or promising son, if different and more truthful views as to such things can be disseminated in the pages of a practical and popular journal like this.

### FISTULAS.

A fistula is an ulcerated channel extending under a surface; hence it may exist in any part of the body. It is sometimes caused in the face by a stoppage of the ducts which convey the saliva from the glands or springs from which it comes; or by some disease in the gland itself; or from an unhealed wound or dead bone. In any case, the cause of the mischief should be ferreted out and the proper remedy promptly applied.

### EYE DEFORMITIES.

One of the most common of these is "strabismus," or squint. It is a want of uniformity in the position and motion of the eyes. The radical cause is the contraction of one of the muscles which move the eyeball. A cure is effected by a division of the tendon of the muscle. The operation is simple, safe and effective, without in any way involving the eye itself or endangering the sight.

The eyelid may be inverted, and the eyelash being in contact with the surface of the eye, inflammation arises, ending in opacity and blindness unless the defect is remedied; or the eyelid may be everted, turned outwards, and come in contact with foreign matter which will damage the sight. Sometimes small tumors are found in the eyelids; these can be easily and promptly removed, and with the most perfect safety.

### WATERY EYES,

Or "Fistula Lachrymalis," is caused by a stoppage of the canal which conveys the tears or water from the eye to the nose, hence the water overflows and runs down the cheek, causing considerable discomfort and inconvenience always, and sometimes inducing irritation and ulceration; this is speedily, easily and perfectly remedied.

When an eye is lost or very much deformed, it can be safely removed and an artificial one substituted, which will give the outward appearance of a good eye.

### NOSE.

The nose may be lost or deformed by internal and external tumors; of the latter variety nasal tumors or polypi are the most common; these fill up the nostril, impede breathing and speaking, and are a source of incessant annoyance; these are readily taken away, and the sense of relief is instantaneous and most agreeable. Sometimes a part, or even the whole of the nose may be wanting or lost by disease, accident, or otherwise. In such cases repairs are made from the adjacent living skin, and a new nose of natural flesh and blood can be re-supplied; and in rare cases, where there is a grievous disfigurement of the face, the misfortune can be remedied in whole or in part by means of skin taken from the arm, and with comparatively little suffering.

A case: A young girl of this city was introduced to the writer; she had lost her nose through an injury; there was no ulceration, the wound having entirely healed. The writer restored the organ last November by building it up from the adjacent skin; the parts healed rapidly, without any unfavorable symptom, she following her usual domestic avocations from the time of the operation, which circumstance, no doubt, facilitated the cure.

In November last the writer restored the tip of the nose of a soldier. A few days later he was consulted by the mother of a young lady for a deformity of the nose of rather a singular character. From a blow received at school, causing inflammation, a portion of the nasal bones came away, resulting in a sinking in of the bridge of the nose, with the tip projecting preternaturally. The daughter was extremely anxious to have an aquiline nose. By the use of an instrument devised and made to meet the case, the patient was rewarded by the angle of the profile of her nose being very much reduced.

### ANAL AND RECTAL DISEASES.

Operations in these cases are comparatively painless and very transient; the aggravating symptoms generally subsiding from the commencement of the treatment. Sometimes it is necessary to divide some bridge of skin or flesh, or remove a slight impediment with the knife; but when this is done, the affair is so really small as hardly to cause a murmur from the most timid. Children may be born with a closed or imperforate anus; the defect is often slight and easily remedied; sometimes it is of so grave a character that a skillful surgeon should be called without an hour's delay.

Foreign bodies sometimes obstruct the passage along the track of the bowels, such as fish-bones, chicken-bones, melon or grape-seed, and the like. Within a week the papers record the death of a man at Troy, N. Y., from inflammation of the bowels, caused, as was found after death, by an oblong stone having lodged crosswise in his bowels; he having been in the habit for two or three years of exhibiting himself to the public for pay, swallowing pebbles with great rapidity, half a dozen on one occasion, one after another, one at a time. The writer was present at one of these exhibitions, about two years ago, near the City Hall of New-York, regarding it at the same time as a disgusting exhibition which the authorities ought to suppress.

Obstructing substances are sometimes introduced from below. The proper remedy is to dilate the bowel, and withdraw the obstruction with appropriate appliances.

### PILES, or Hæmorrhoids, is the most common of all rectal diseases. The urgency of the symptoms varies with the character of the malady. Piles are small blood-tumors near the edge and extremity of the lower bowel, and are caused by the enlargement of the blood-vessels of the parts. Piles are internal or external. The internal are tumors varying in size from a pea to a walnut or larger; they are of a dark brownish or bright red color, according to the degree of inflammation present; they cause great inconvenience, and sometimes most acute suffering at every evacuation. Some persons are totally prostrated for two or three hours afterwards, lying on the bed in the mean while in a state of great suffering, the thickened and vascular condition of the inner lining of the parts (the mucous membrane) being exceedingly liable to bleed from straining and pressure.

TO BE CONTINUED.

